# The Health and economic effects of light rail lines: design, methods, and protocol for a natural experiment

**DOI:** 10.1186/s12889-019-6518-6

**Published:** 2019-02-15

**Authors:** Lawrence D. Frank, Jennifer L. Kuntz, James E. Chapman, Eric H. Fox, John F. Dickerson, Richard T. Meenan, Brian E. Saelens, Deborah R. Young, Janne Boone-Heinonen, Stephen P. Fortmann

**Affiliations:** 1 Urban Design 4 Health, Inc., Rochester, NY USA; 2 Center for Health Research, Kaiser Permanente Northwest, Oregon, Portland USA; 3 Health & Community Design Lab, Schools of Population and Public Health and Community and Regional Planning, University of British Columbia, Vancouver, BC Canada; 4 Seattle Children’s Research Institute and the University of Washington, Seattle, WA USA; 5 Center for Research & Evaluation, Kaiser Permanente Southern California, Pasadena, CA USA; 6 Oregon Health & Science University, School of Public Health, Oregon, Portland USA

**Keywords:** Transportation, Light rail transit, Built environment, Active travel, Physical activity, Health care utilization, Environmental measurement methods

## Abstract

**Background:**

The health impacts of community design have been studied extensively over the past two decades. In particular, public transportation use is associated with more walking between transit stops and shops, work, home and other destinations. Change in transit access has been linked with physical activity and obesity but seldom to health outcomes and associated costs, especially within a causal framework. Health related fiscal impacts of transit investment should be a key consideration in major transit investment decisions.

**Methods:**

The Rails & Health study is a natural experiment evaluating changes in clinical measures, health care utilization and health care costs among Kaiser Permanente Northwest (KPNW) members following the opening of a new light rail transit (LRT) line in Portland, Oregon. The study is prospectively following 3036 adults exposed to the new LRT line and a similar cohort of 4386 adults who do not live close to the new line. Individual-level outcomes and covariates are extracted from the electronic medical record at KPNW, including member demographics and comorbidities, blood pressure, body mass index, lipids, glycosylated hemoglobin, and health care utilization and costs. In addition, participants are surveyed about additional demographics, travel patterns, physical activity (PA), and perceived neighborhood walkability. In a subsample of the study population, we are collecting direct measures of travel-related behavior—physical activity (accelerometry), global positioning system (GPS) tracking, and travel diaries—to document mechanisms responsible for observed changes in health outcomes and cost. Comprehensive measures of the built environment at baseline and after rail construction are also collected. Statistical analyses will (1) examine the effects of opening a new LRT line on chronic disease indicators, health care utilization, and health care costs and (2) evaluate the degree to which observed effects of the LRT line on health measures and costs are mediated by changes in total and transportation-associated PA.

**Discussion:**

The results of the Rails & Health study will provide urban planners, transportation engineers, health practitioners, developers, and decision makers with critical information needed to document how transit investments impact population health and related costs.

## Background

Rates of overweight and obesity have steadily risen in the last half-century and obesity is now one of the largest modifiable health risk factors for numerous common diseases, including diabetes, hypertension, cardiometabolic disorders, cancer, asthma, depression, and musculoskeletal disorders. [1–9] Nearly three-quarters of U.S. adults are now overweight or obese and the expected future increase in obesity rates will lead to millions of additional cases of diabetes, cardiovascular disorders, and other associated diseases. [2, 10, 11] These trends will also lead to marked increases in health care costs. [12] In turn, regular physical activity (PA) reduces the risks of obesity, diabetes, and associated disorders. [1, 13] The high prevalence of physical inactivity in the U.S. is a major cause of high chronic disease rates and results in a large population-attributable risk from physical inactivity. [14–16]

Because these health problems are partially attributable to sedentary lifestyles, a promising area of research is now focusing on measuring the health impacts of the built and natural environment where people live and work. Researchers are investigating the population-level health impacts of changes in travel patterns resulting from transportation investments and land use patterns or local “walkability,” [17–21] and have demonstrated that transportation investments, land use patterns, and access to open space can impact physical activity and obesity prevalence. [22–38]

Growing evidence of the role of community design and the built environment in stimulating an active lifestyle has led to an increase in environmental interventions intended to promote PA. [39] However, studies that examine the impact of these interventions have rarely captured objective measures of PA or assessed the relationship between built environment and health-related biomarkers such as body mass index (BMI), blood pressure, or glycemic control. [40–42] Available studies also do not capture the behavioral and personal factors within a possible “causal pathway” that may mediate the relationship between the built environment and health outcomes. Thus, detailed, longitudinal data are needed, first to fully establish the relationship between improvements in community design—including public transportation infrastructure—and objectively measured clinical health outcomes; second, to determine how behavioral factors, specifically changes in physical activity, influence and mediate this relationship; and finally, to understand the impact environmental interventions such as transit investments have on healthcare utilization and costs. This information will allow policymakers and other decision makers to best align limited infrastructure investment resources with their potential for improved population health and cost savings.

The Rails & Health study is a longitudinal natural experiment that examines the impact of a major public transportation infrastructure improvement—the opening of a new light rail transit (LRT) line—on clinical health outcomes and health care utilization and costs. The study seeks to leverage unique and robust observational research data to advance our understanding of the multi-faceted impacts of large-scale transit investments on public health. This article describes in detail the Rails & Health study design, including participant recruitment, an extensive objectively-measured and self-reported health and environmental data acquisition effort, measurement methods, and statistical analyses. We also present strategies used to establish consistent, participant-specific neighborhood environmental measures related to community walkability, access to destinations and services, and social contexts.

## Methods

### Rails & Health study design overview

**Fig. 1:**
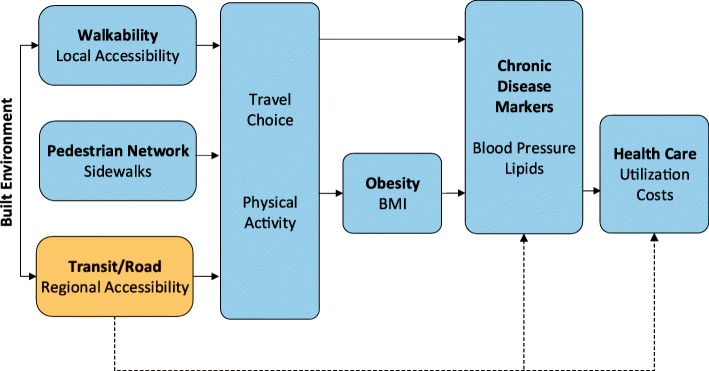
Conceptual Model.©2019 Kaiser Permanente Center for Health Research

The primary aim of the Rails & Health Study is to examine the impact of opening a new LRT line on chronic disease indicators, health care utilization, and health care costs. The case-control design compares 3036 adults who live near the rail line to 4386 adults with similar demographic characteristics who live in similar areas away from the rail line (Fig. 1). To address this aim, measures of health care utilization and costs for the three years before and three years after the line opened are extracted from the KPNW electronic medical record (EMR). Chronic disease indicators include blood pressure, BMI, lipids measured by non-HDL cholesterol, and glycosylated hemoglobin (HgbA1c).

The second aim of the study is to evaluate the degree to which the observed impact of the LRT line on chronic disease indicators and health care utilization and costs are mediated by changes in total and transportation-associated PA (Fig. 1). To address this question, we will analyze outcome data from a subset of 600 participants—including objectively measured metrics of PA gathered using accelerometry, global position system (GPS) tracking, and self-report travel diaries, as well as a transportation and neighborhood perceptions survey. Collecting objective built and micro-scale pedestrian environment measures will allow us to evaluate how that environment may influence changes in PA, clinical measures, and health care costs.

### Study setting

The study evaluates the impact of the Portland–Milwaukie Light Rail Project, also known as the Orange Line, which opened in September 2015. The Orange Line is a 7.3-mile, 10-station addition to the 53-mile, 87-station Metropolitan Area Express (MAX) light rail system of the Tri-County Metropolitan Transportation District of Oregon (TriMet). TriMet is the public agency that operates mass transit in the Portland, Oregon metropolitan area. The new line connects downtown Portland to the City of Milwaukie, directly to the south, and includes the first multi-modal, car-free river bridge in the U.S. and concurrent improvements to pedestrian infrastructure within LRT station areas.

### Participants and recruitment

Study participants are members of Kaiser Permanente Northwest (KPNW), an integrated health care system that provides ambulatory and hospital care to more than 600,000 patient-members in northwest Oregon and southwest Washington. Critical to this study, KPNW uses a comprehensive EMR, member addresses are updated frequently, and historical addresses are maintained.

### Overall cohort (aim 1)

**Fig. 2:**
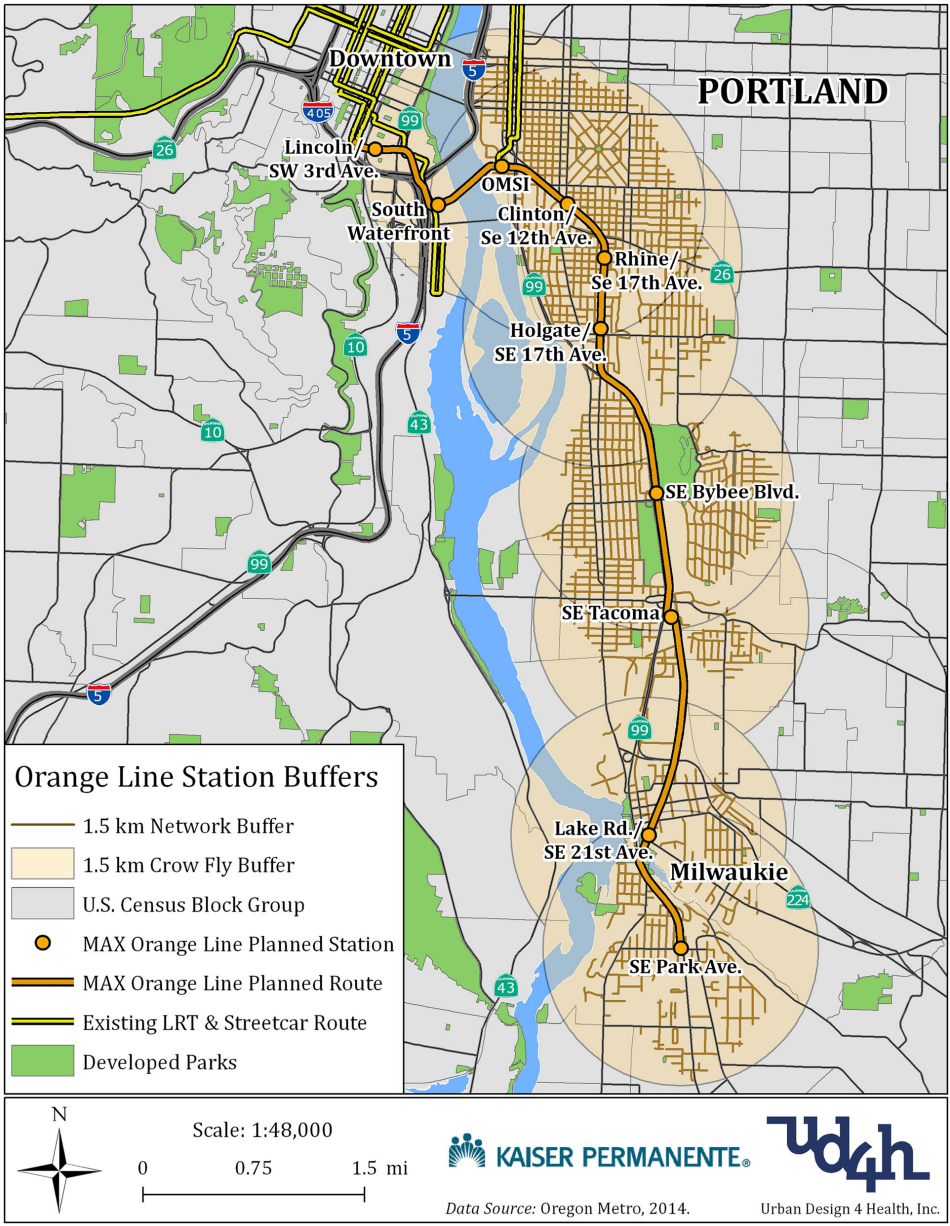
Map illustrating 1.5-km walkable road network and “crow-fly” proximity buffers around MAX Orange Line station areas used for participant recruitment.©2019 Urban Design 4 Health, Inc.

Participants in the overall cohort are aged 18–74 years and have lived at their current address for at least three years prior to the LRT line opening in September 2015. From among the pool of eligible participants, cases were identified as participants who had residential addresses in census block groups within a 1.5-km road network buffer of one of the new LRT stations on the Orange Line (Fig. 2). Controls had residential addresses in census block groups with a similar baseline census median household income category and similar regional accessibility (defined by transit peak-hour travel time to downtown Portland) to cases. However, controls’ block groups did not intersect a 1.5-km road network buffer of any existing rail station. Residential address was the most recent address included in KPNW membership data. These data are updated and archived during almost every health care contact. The residence address of each participant was also geocoded to exact latitude and longitude and to the U.S. Census block, ZIP code, and county using a standard Geographic Information System (GIS) application. These were further validated using the Google Maps Application Programming Interface. Participant locations that were not precisely matched using the geocoded tool were investigated and manually adjusted to the actual physical address.

Demographic characteristics, clinical measures, and healthcare utilization data for members of the overall cohort are obtained through the KPNW EMR. Members of the overall cohort were also invited to complete an online survey regarding their transportation and neighborhood perceptions before September 2015. The invitation email contained a link to a survey website, which provided details about the research study and elicited consent for participation. Participants who completed this initial survey were asked to also complete a follow-up survey after the opening of the LRT line. Participants were entered into a drawing for a $100 gift card each time they completed the survey.

### Behavioral cohort (aim 2)

A subset of the overall cohort—600 members— was recruited and asked to collect objective PA and travel patterns over two seven-day periods: prior to and one year after the opening of the LRT line. This data collection included completion of a travel diary and PA monitoring using an accelerometer and GPS device. All participants in this “behavioral cohort” also completed the online transportation and neighborhood perceptions survey.

To recruit this subset of participants, we stratified cases into four categories based on how distant each case’s residence was from an LRT station. Recruitment of cases started in block groups immediately adjacent to each Orange Line station and worked outward until the LRT line opened, ending the recruitment window. Controls were recruited by randomly selecting members from the matched control block groups. Potential participants were sent an introductory email with a description of the study and a link to the online survey, and then were contacted by telephone. During the telephone call, study staff explained the study procedures, confirmed the participant’s current address, and obtained verbal consent for initial and follow-up data collection. Participants were then mailed a paper copy of the consent document, the accelerometer and GPS devices, a paper travel diary, written instructions, and materials for returning the devices and travel diary. All participants received a follow-up phone call after receiving the study materials to explain how to use the devices, answer questions, and encourage study completion. Participants who completed all data collection at both time points received a $75 gift card.

### Data collection

Table 1 includes an overview of study data collection for the overall cohort and the behavioral cohort. We collected EMR data for all participants, data from a transportation and neighborhood perceptions survey for a subset of the overall cohort and all members of the behavioral cohort, and detailed transportation and activity data from the behavioral cohort. Additionally, we constructed comprehensive measures to describe the macro scale (walkability) and micro scale (pedestrian) environment of participants’ neighborhoods. Pedestrian environments were assessed for both home and work locations for the behavioral cohort.

**Table 1:** Summary of Data Collection for Study Population Sub-groups

	Study Groups		Data Source and Measurement		
	Overall Cohort	Behavioral Cohort	Environmental Data	KP EMR	Measurement Method and/or Time Frame
OUTCOME MEASURES					All 3 years pre- and post-light rail transit (LRT)
Medical Care Costs	X			X	
Body Mass Index	X			X	
Blood pressure	X			X	
Laboratory Data (lipids, HgbA1c)	X			X	
INDIVIDUAL COVARIATES					
Age, sex	X			X	
Race, ethnicity	X			X	
Smoking, alcohol use	X			X	
Chronic disease/co-morbidities (diagnoses)	X			X	Charlson Index [44], 3 years pre- and post- LRT
Medication use	X			X	3 years pre- and post- LRT
TRANSPORTATION AND NEIGHBORHOOD PERCEPTIONS SURVEY VARIABLES	*				All baseline and 1-year post-LRT
Race/ethnicity details, income, education	*	X			Census Items
Functional Status	*	X			10-item scale from PROMIS [45, 46]
Duration of residence prior to enrollment	*	X			Prior 3 addresses
Residential Preference	*	X			MetroAtlanta Pref Survey
Perceived Walkability	*	X			
Transportation Use	*	X			
Household Environment	*	X			Occupants, exercise equipment, pets
Worksite Environment	*	X			Address, exercise promotion [49, 50]†
PHYSICAL ACTIVITY AND TRANSPORTATION MEASURES					At baseline and 1-year post-LRT
Accelerometry		X			7-Day Actigraph® GT3X+,
GPS tracking		X			7-day BT Q1000XT GPS
Travel Diary		X			7-day Modified 2009 NTHS [54]
BUILT ENVIRONMENT VARIABLES					
Neighborhood walkability		X	X		
Regional transportation accessibility*		X	X		
Pedestrian Landscape		X	X		MAPS tool [66]
Park Access		X	X		

NOTE: Participants in the behavioral cohort are included in the overall cohort

*For a subset of the overall cohort

The Kaiser Permanente Northwest Institutional Review Board reviewed and approved all study procedures and materials. Informed consent and Health Insurance Portability and Accountability Act authorization were waived for EMR data collection. Participants who completed surveys provided consent at the time of survey initiation after reading the consent language, while participants undergoing behavioral data collection provided verbal consent and authorization. Because we used telephone recruitment and remote data collection (no direct visit), signed consent was waived for the behavioral cohort.

### Electronic medical record-derived measurements

All demographic and clinical covariate data, clinical outcome data, and health care utilization data were extracted directly from the KPNW EMR (an instance of Epic®). These measures were collected for the overall cohort for the three years before and after the LRT line opened.

The clinical outcomes for the primary study aim obtained from EMR laboratory and encounter data are blood pressure, BMI, non-HDL cholesterol, and HgbA1c. Health care utilization is collected from EMR encounter data and includes average monthly outpatient visits, average monthly length of inpatient stays, and average total monthly medication utilization. Pharmacy dispensing data are also collected from the EMR since KPNW includes a closed pharmacy benefit. Health care costs are calculated using a Standardized Medical Care Costing Model developed at the Center for Health Research to account for all services, procedures, and products received by a member, as captured in the KPNW data systems. [43] Demographic data collected from the EMR include birthdate (age) and self-reported sex, race, and ethnicity. Income and education data are not complete in the EMR, so census-derived values were used. Clinical covariate data included history of smoking and alcohol use and the presence of chronic diseases and comorbidities, identified through diagnosis codes assigned during health care encounters and aggregated using the Charlson Comorbidity Index. [44]

### Transportation and neighborhood perceptions survey

The transportation and neighborhood perceptions survey collected additional information about transportation use, perceived PA, and perceived neighborhood characteristics prior to and after the opening of the LRT line (survey available from the authors). These data will be used in descriptive analyses and as covariates in models that examine whether potential changes in clinical, utilization, and cost outcomes related to the LRT line are impacted by PA and transit use. The survey includes self-report measures of: (1) demographics, including income, education, race, ethnicity and social habits (e.g., smoking, alcohol use); (2) physical functioning, or functional status, as measured through a validated 10-item short form recommended by the NIH Patient-reported outcomes system (PROMIS) [45, 46]; (3) duration of residence prior to study enrollment and prior three addresses; (4) preferences for pedestrian- and transit-oriented neighborhoods versus auto-oriented neighborhoods, measured through an established series of stated preference survey questions [47]; (5) perceived walkability of a participant’s neighborhood, as measured through the abbreviated Neighborhood Environment Walkability Scale (NEWS-A) [48]; (6) typical travel behavior and travel modes to common destinations (e.g., work, shopping, recreation), travel times, costs, and factors affecting mode choices; (7) household factors that may influence PA, such as the presence of children, elderly dependents, pets, and exercise equipment; (8) perceptions and opinions about mass transit systems and use; and, (9) worksite environment, including a participant’s primary worksite address and perceptions of worksite exercise and health promotion activities. [49, 50]

### PA and transportation data collected from behavioral cohort

#### Accelerometry

Physical activity was objectively measured with GT3X+ accelerometers (Actigraph®, Pensacola, FL). The GT3X+ is a triaxial monitor that detects acceleration in the vertical, anteroposterior, and mediolateral axes. [51] Participants were asked to wear a monitor during all waking hours for seven consecutive days, and also to record times when they were not wearing the monitor. Accelerometry data were collected and stored in 15-s intervals. The data were categorized into physical activity bouts, defined as time intervals having > 500 accelerometer counts per 30-s epoch (cpe) for at least seven minutes, allowing for up to two minutes of epochs below that threshold during the seven-minute interval. The threshold of 500 cpe was chosen to capture light PA that might be associated with slow walking at an average speed of 3 km per hour. [52, 53]

#### Global positioning system (GPS) monitoring

Geographic position and instantaneous movement speed provide contextual information about PA behaviors such as the location of an activity and the mode of transportation. These data were collected using the BT Q1000XT GPS Travel Recorder (QStarz, Taipei, Taiwan). Participants were asked to wear or carry the portable travel recorder for seven days, concurrent with accelerometer use and travel diary data collection. The device can be attached to the accelerometer belt or another belt and works up to twenty hours when set at 15-s epoch acquisition.

#### Travel diary

Participants in the behavioral cohort also completed a written travel diary for each day of accelerometer and GPS wear. The travel diary is based on the 2009 National Highway Transportation Survey, with additional questions about recreational travel. [54] Participants are instructed to log departure and arrival locations and times, travel mode, trip purpose, and destination activities for all trips made within the 7-day period. [55]

#### Integration of accelerometer, GPS, and travel diary data

Data from the GPS and accelerometer units were cleaned, processed, and merged using the All-in-one Spatial Activity Processor, an open source tool developed by researchers at Portland State University. [56] The tool identifies erroneous GPS data points (e.g., unrealistic speed, distance traveled, or position) and smooths the data using automated and systematized procedures. [57] GPS records were also plotted using spatial GIS software and spot-checked to identify any other systematic errors.

Once GPS and accelerometer data were merged, discrete trips were differentiated using methods that examine speed variations and time spent at specific destinations. Travel mode (e.g. walking, biking, mass transit, automobile) was ascertained based on travel speed, route, and transportation network characteristics. [58] Individual trips and tours were constructed to capture travel between destinations. [59] Cluster detection methods were employed to identify points centered on a specific location and occurring over a period of at least 120 s, which indicates a destination.

Travel diary information was entered into a database and algorithms were used to identify missing and possibly erroneous data prior to merging it with GPS and accelerometer data. Trip records were flagged for any of the following errors: trip ended before it started; trip departed before the preceding trip arrived; trip spanned more than 12 h. Research staff contacted participants as needed to fill in missing entries or to clarify erroneous entries.

Merged accelerometer and GPS data were then joined to travel diary data by matching the arrival/departure times from the travel diary with the closest time stamp in the merged GPS and accelerometer data. Collectively, the data integration allows for validation of behaviors across data streams, provides additional information for data cleaning and imputation, and facilitates the derivation of summary PA metrics.

### Measures of built environment

We derived comprehensive measures of the built environment for each participant’s residence and workplace address at baseline. We will test how these measures—including neighborhood walkability; regional accessibility to destinations; seating, lighting, sidewalks, and other design features; and access to open space, moderate the effect of the LRT line opening on PA, clinical health measures, and costs.

### Pedestrian-enhanced walkable road network

As a formative step in the creation of built environment measures, we assembled a comprehensive network that pedestrians can traverse, including roadways and non-motorized, multi-use pathways. This walkable network was used for three primary purposes: (1) defining the catchment area a participant can walk to within a given distance (street network buffer) from their home and their work, (2) determining the locations of intersections and cul-de-sacs, and (3) determining the network distance and walking travel times, from a participant’s home and work, to the nearest work and non-work destinations.

**Fig. 3:**
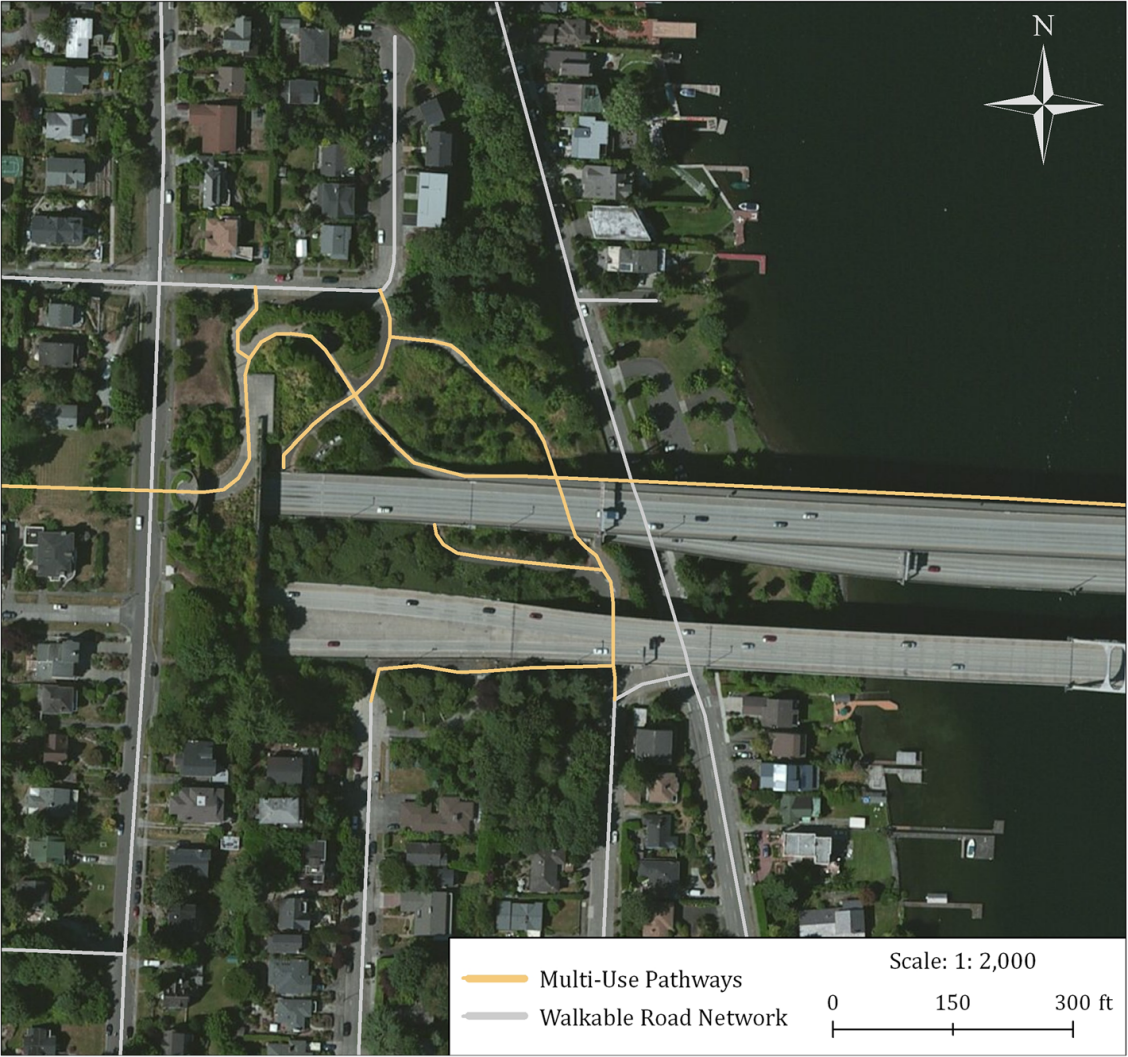
Example section of the “pedestrian-enhanced” walkable road network that excludes freeway road segments while integrating multi-use pathways.©2019 Urban Design 4 Health, Inc

We acquired a road centerline file and information describing pedestrian and cyclist non-motorized pathways from Metro, the metropolitan planning organization serving the Portland Region. [60] All road types where pedestrians are not permitted—including interstates, freeways, and other limited access ramps and interchanges—were removed to create a “walkable road network.” Non-motorized facilities such as regional and local trails, park pathways, and cul-de-sacs/dead-end cut throughs were also included (Fig. 3).

### Regional accessibility

Regional accessibility measures relative travel time and distance, by location and mode, to major, regionally-significant destinations. It is derived from regional travel demand models used to inform transportation investment decisions and is based on the trip distribution sub-model that predicts where people will go for given types of trips. It captures access to jobs, parks, shops, and other destinations within a given amount of time. The study used locations of regional significance defined by the Metro 2040 Growth Concept. [60] These locations were considered to be the most important regional centers and were targeted for concentrated future growth.

The measures of regional accessibility included the network distance and estimated travel times (for both single occupancy vehicles and on transit) between all participant origin locations and regionally significant destinations, as well as an average measure of the network distance to these destinations. Auto travel time estimates were obtained from Metro using their modeled “centroid based” zone to zone travel times from the regional travel demand model. Zone to zone private vehicle travel times reflect actual or “congested” travel speeds for peak and off-peak periods. Transit travel time estimates for peak and off-peak (including walking to and from the transit system) were calculated using General Transit Feed Specification transit schedule data (GTFS Data Exchange: http://transitfeeds.com).

### Walkability

Walkability combines measures of proximity (density and land use mix), street connectivity or route directness, and retail floor area ratio or street setback, forming a composite measure of local accessibility. Walkability is a validated construct that captures variations in development patterns that are associated with behavior, including travel and PA. We focused on walkability variables that most influence choices about transportation mode (e.g., driving vs. walking). [61]

### Participant network buffers

**Fig. 4:**
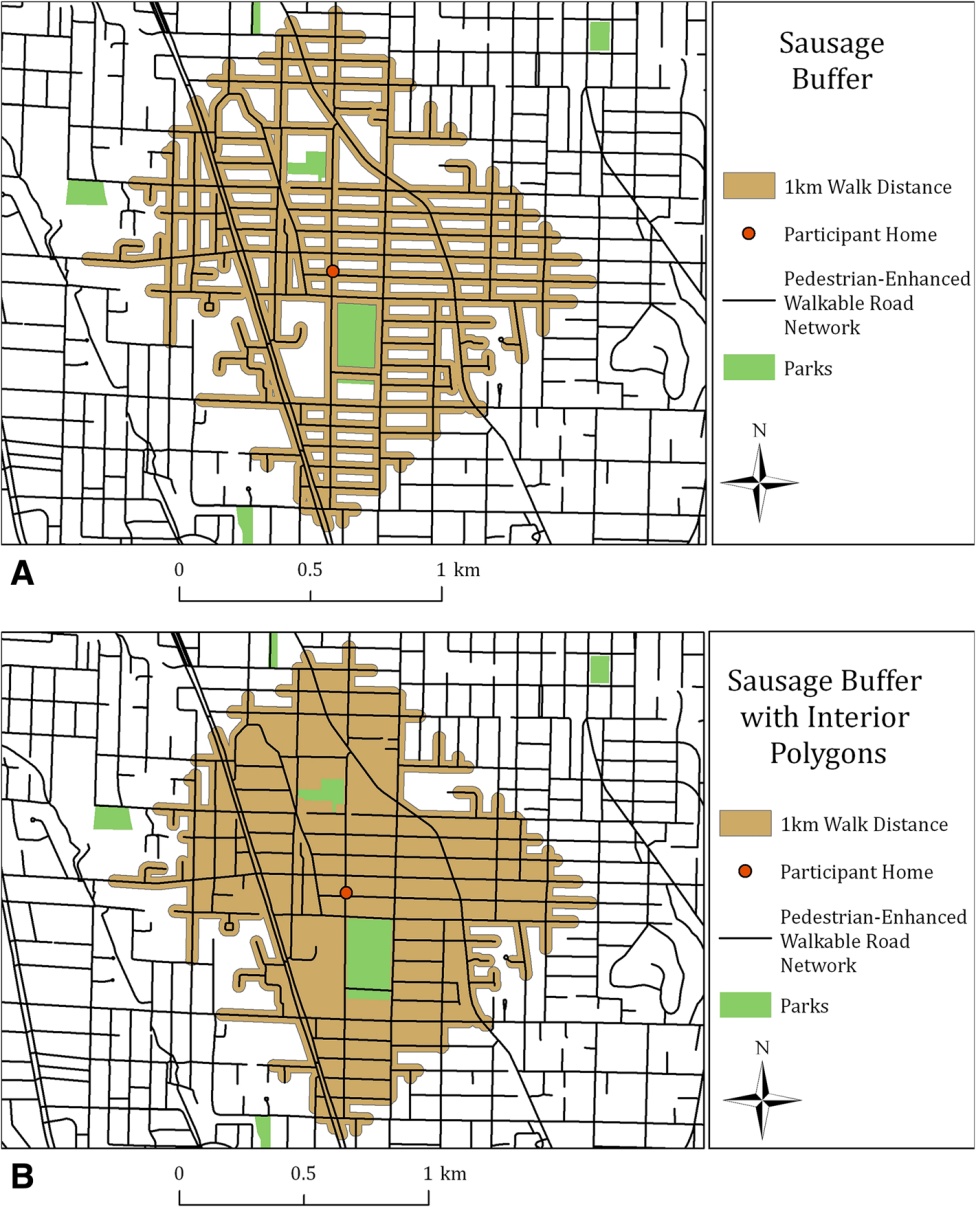
Buffer comparison showing the “sausage” or balloon buffer and the same buffer combined with the interior or island polygons. Image A is the buffer form used to calculate all built environment variables including counts and intersecting features. The area derived from the Image B form was used as the denominator for all density measures.©2019 Urban Design 4 Health, Inc

Spatial catchment areas, referred to here as buffers, originating at a participant’s home or work address, capture the area a participant can access on the road network for a given distance. State of the art “sausage” based network buffers (see Fig. 4) were developed to delineate areas within a 1-km (0.6 mile) walk distance, with a 25-m (82 ft) trim or setback from the roadway. [42, 62–64] The sausage buffer first defines the catchment area along a 1-km distance in all directions on the network. The 25-m trim distance from the road network removes areas not accessible to pedestrians set back from the road network. Our sausage buffer design also supports specific requirements of each built environment measure. For example, it nets out non-residential areas to create the land area denominator for calculating net density measures (see Fig. 4).

### Pedestrian landscape

The Microscale Audit of Pedestrian Landscape (MAPS) tool is used to quantify microscale characteristics of street segments shown to have relevance for walking and other forms of PA. [65] Routes between home and work for each participant were characterized using the MAPS tool. [66, 67] The MAPS tool accounts for a variety of street design features, including speed limit, sidewalk quality, land use, transit service, street crossing environment, bike facilities, landscaped buffers, building characteristics, crosswalks, crossing width, and signalization. [68] Based on these features, the tool provides distinct scores for overall route, street segments, intersections, and cul-de-sacs. Overall, 47 route-related variables, 24 segment-related variables, 21 crossing-related variables, and six cul-de-sac-related variables were assessed and aggregated into 11 subscale, overall, and cross-sectional variables based on expert consensus, theoretical assumptions of how the built environment influences physical activity, and policy-relevant considerations. [69]

### Park environment

Public parks offer residents opportunities for physical activity among other health benefits. We counted the number of parks and calculated total park area intersecting and accessible within the 1-km sausage buffer around each participant’s residence and work location, as well as the distance from each residence and work location to the nearest park. We included only developed, public parks with regular municipal maintenance, excluding natural areas not meant for regular access by the public and private areas such as golf courses. We separated parks into three size categories: (1) small park, parklet, or pocket park (< 1 acre), (2) medium park (1–50 acres), and (3) large park or state park (> 50 acres).

### Crime

Crime is a significant factor impacting the perceived safety and comfort of walking in a neighborhood. Crime data were obtained from the uniform incident reporting system used by the Portland Bureau of Police tracking all crime offenses. Crimes were separated into violent and non-violent. Violent crime consisted of homicide, rape, aggravated assault, kidnapping, robbery and arson with all remaining crimes identified as non-violent, including motor vehicle theft, fraud, prostitution, driving under the influence and drug related incidents. Geographic locations of each crime incident were plotted and the counts of crime incidents within each participant’s home and work buffer were summed to provide a measure of neighborhood crime.

### Statistical analyses plan

#### Aim 1 analyses

Our first aim is to assess the impact of the LRT line opening on clinical health outcomes, health care utilization, and health care costs from three years before to three years after the line opened. These analyses include participants in the overall cohort who have at least four outcome measures over the entire six-year period, with at least one of each measure occurring prior to and after the LRT line opening.

### Clinical health outcomes

Multilevel modeling will be used to determine the effect of the LRT line opening on clinical health measures – including blood pressure, BMI, blood lipids, and HbgA1c. Time will form the first level of the model and all available measurements for each participant will be included. Person will form the second level of the model that will include person-level covariates of age, sex, and comorbid conditions measured at the earliest available time point. Since people are nested within block group, block group will form the third level of the model with a dummy variable for case versus control block group. This model will estimate a trajectory of the outcomes over time for each participant and will examine differences in trajectory based on case or control status.

### Health care utilization and costs

Segmented regression analysis will be used to compare trends in health care utilization and costs for case and control groups before and after the LRT line opening. Ultimately, this method allows us to statistically assess how much the introduction of the LRT line changed health care utilization and cost immediately and over time; transiently or persistently; and instantly or with delay. Specifically, we will perform a basic segmented regression analysis in which the overall six-year time period is divided into pre- and post-intervention segments. For each segment, separate intercepts and slopes will be estimated, thus allowing for statistical tests of changes in intercepts and slopes pre- to post-intervention. Statistical power will be enhanced if we find stable pre-intervention trends in the dependent variable of interest. We will use month as the unit of analysis (36 months pre- and 36 months post-LRT introduction). Health care utilization and cost measures will include average monthly outpatient visits and costs, average monthly inpatient length of stays and cost, average total monthly medication cost, and average total monthly health care expenditures.

### Aim 2 analyses

Structural equation modeling (SEM) will be used to determine the degree to which changes in clinical outcomes and health care costs are mediated by changes in total and transportation-associated PA (measured through accelerometer, GPS, and travel diary data), when controlling for built environment measures such as walkability and measures of pedestrian and recreational infrastructure. SEM allows us to test the relationships among the variables simultaneously. To accomplish this, change scores from the first time point (2–3 years prior to the opening of the LRT line) to the last time point (2–3 years after the opening) for all variables will be examined. To test for mediation, a multilevel structural equation modeling approach will be used. Potential moderators of the effect of the new rail line on health outcomes will also be explored. For example, the interaction between case or control status and variables such as age, sex, race/ethnicity, distance from home to station, baseline walkability, pedestrian network (as measured by the MAPS tool), and baseline functional status will be incorporated into multilevel models to test if the degree of impact of the new rail lines varies across the levels of these potential moderator variables.

### Sample size and power

The sample size for this natural experiment was fixed and determined by the number of KPNW members who had residential addresses in census block groups within a 1.5-km road network buffer of one of the new LRT stations. These cases were matched at the block group level to an equivalent number of members living outside of a 1.5-km road network buffer of any existing rail station. During the planning phase of the study, the population anticipated to have sufficient data for our clinical outcomes – based on estimated variations in the data capture of clinical health outcome measurement and loss to follow-up – was 3050 participants for BMI and blood pressure; 2350 for blood lipids; and 850 for HbgA1c. The detectable changes between cases and controls for the main outcome measures were calculated with statistical power of 0.80 and an alpha level of 0.05. The calculations of detectable differential change used means and standard deviations for the outcome measures as estimates of the baseline values, accounted for the correlation between individuals nested within block groups, and assumed no change in the control group. Given these parameters, we estimated that our study would detect significant differences between cases and controls if the change over time in the light rail group was 4% greater for BMI, 1.4% greater for BP, 2.8% greater for HgbA1c, and 2.4% greater for non-HDL cholesterol. These levels would be clinically significant at the population level.

For our analysis of healthcare costs, interrupted time series analyses are considered to have good statistical power if the trend prior to the system change is fairly stable. Similar segmented regression analyses have had adequate power to detect modest effects and were used to inform policy at the system level using data for 12 time points pre- and post-intervention. [70, 71] Our study captures six total years of healthcare cost data, or 72 time points, which provides adequate power.

## Discussion

Recent cross-sectional evidence makes clear connections between built environment, activity patterns, and health outcomes; increasing levels of transit access and walkability are associated with increased physical activity and reduced levels of chronic disease. [72] These findings have been extended to health care costs using costs of illness methods in a few limited unpublished studies. [73] So far, no research has shown how clinically assessed, EMR-derived health outcomes and associated health care utilization and costs relate to the built environment. Further, no research we are aware of has directly connected built environment data with EMR data containing clinically assessed health outcomes and related costs within the framework of a longitudinal intervention designed to assess causation.

The methods we describe constitute a unique research design to better understand the potential causal impacts of transportation investments and land use on physical activity, obesity, and clinically assessed health outcomes and cost. The ability to connect multi-billion-dollar transportation investments with similarly-scaled, currently externalized health care costs holds considerable research promise. The results from this study and others that may build on it could have a major influence on transportation investment decision-making protocols. Cost-benefit tools currently used to justify transportation investments do not account for the health impacts and costs they incur, resulting in decisions that promote roadway improvements over transit and non-motorized investments. Internalizing these costs within the transportation investment process has the potential to better estimate the collective societal impacts of these major investments.

Sedentary and active travel choices are made based on the quality, convenience, and relative utility of available travel options. [74, 75] It can be argued that mode choice roughly follows the distribution of funding spent per mode over the past several decades. Mounting health care costs from chronic diseases within the context of an aging population warrant critical attention to underlying built environment factors that shape travel patterns and in turn impact population health.

The Rails & Health study will provide information that documents how a new transportation option can alter PA, health, and health care costs over time. This study will provide valuable data and contribute unique methods. It may also present a compelling rationale for cost benefit-tools and empirically grounded health impact assessments. These approaches can collectively assess health outcomes and costs stemming from contrasting transportation investment and land development options.

## Abbreviations

BMI: Body Mass Index
EMR: Electronic Medical Record
GIS: Geographic Information System
GPS: Global Positioning System
KPNW: Kaiser Permanente Northwest
LRT: Light Rail Transit
MAPS: Microscale Audit of Pedestrian Streetscapes
PA: Physical Activity
SEM: Structural Equation Modeling
TriMet: Tri-County Metropolitan Transportation District of Oregon
